# On power and its corrupting effects: the effects of power on human behavior and the limits of accountability systems

**DOI:** 10.1080/19420889.2023.2246793

**Published:** 2023-08-24

**Authors:** Tobore Onojighofia Tobore

**Affiliations:** Independent Scholar, Yardley, PA, USA

**Keywords:** Dominance, Dominance Hierarchy, high power and low status, power, power addiction, power and aggressive behavior, power and ambition, power and bariatric surgery, power and bias, power and cooperation, power and corruption, power and credibility, power and dehumanizing behavior, power and demeaning behavior, power and disinhibited behavior, power and entitlement, power and gossip, power and hypocrisy, power and overconfidence, power and physical attractiveness, power and self righteousness, Power and Sex, power and sexual harassment, power and unethical behavior, power and victimhood, Power and self-interested behavior, structural limits of accountability systems, Power and Access, Power and Nepotism, Social Rank, Social Status, Socioeconomic status, Power and Status, Power and Mental Health, Powerlessness and behavior, Power and size, Power and Evolution, Justice systems

## Abstract

Power is an all-pervasive, and fundamental force in human relationships and plays a valuable role in social, political, and economic interactions. Power differences are important in social groups in enhancing group functioning. Most people want to have power and there are many benefits to having power. However, power is a corrupting force and this has been a topic of interest for centuries to scholars from Plato to Lord Acton. Even with increased knowledge of power’s corrupting effect and safeguards put in place to counteract such tendencies, power abuse remains rampant in society suggesting that the full extent of this effect is not well understood. In this paper, an effort is made to improve understanding of power’s corrupting effects on human behavior through an integrated and comprehensive synthesis of the neurological, sociological, physiological, and psychological literature on power. The structural limits of justice systems’ capability to hold powerful people accountable are also discussed.

## 1. Introduction

Scholars across different disciplines have tried to define power [1]. It has been defined as having the potential to influence others or having asymmetric dominion over valuable resources in a social relationship [2,3]. It has also been defined as the capacity of people to summon means and resources to achieve ends [1]. In addition, it has been described as having the disposition and means to asymmetrically impose one’s will over others and entities [4]. Taken together, power can be defined as being able to influence others due to asymmetric dominion of resources, the capability to summon means to achieve ends, and being able to impose one’s will over others and entities. Power is an all-pervasive and fundamental force in human relationships and plays a valuable role in social, political, and economic interactions [4]. It plays an important role in many aspects of human life, from the workplace, and romantic relationships, to the family [5,6]. Power is dynamic, and it resides in the social context, and should the social context change, power relations tend to change as well [1]. There are different types of power and their effective utility lies within a limited range [7].

Power differences within groups enhance group functioning by promoting cooperation [8], creating and maintaining order, and facilitating coordination [9]. Most people want to have power and there are many benefits to having power. People desire power to be masters of their own lives and to have greater autonomy over their fate [10,11]. Position in the dominance hierarchy is correlated with both general and mental health [12] and associated with reproductive access, grooming from others as well as preferential food and spaces [13]. Elevated power promotes authentic self-expression [14], reduced anger, greater happiness, and positive emotions/mood [5]. In contrast, low power is associated with negative emotions (discomfort and fear) [15–17], increased stress, and alcohol abuse [18].

Evolutionarily, dominance and perceptions of power cues are associated with body size. Indeed, social status can be attained through two pathways: prestige or dominance [13]. Height is positively related to dominant status [19]. High-status prestigious and dominant individuals tend to be judged as taller, and taller individuals as higher in prestige and dominance [20]. Also, dominant high-status people tend to be judged as more well-built, and more well built individuals as dominant [20]. Power and status (i.e., respect and admiration) represent different dimensions of social hierarchy but are positively correlated [21]. Power is causally connected to status because power can lead to the possession of status and status can result in the acquisition of power [21]. Power from social status is a central and omnipresent feature of human life and they are both correlated in terms of control of institutions, political influence, material resources, and access to essential commodities [22,23]. From an evolutionary perspective, high status is sought because reproductively relevant resources, including territory, food, mating opportunities, etc. tend to flow to those high in status compared to those low in status [24].

Having power affects the human body physiologically, neurologically, and psychologically. Power is linked with neurological alterations in the brain. Indeed, power triggers the behavioral approach system [2,25] while powerlessness undermines executive functioning [17]. Low social power state compared to high or neutral power is associated with significantly reduced left-frontal cortical activity [26]. Animals research suggests that dominance status modulates activities in dopaminergic neural pathways linked with motivation [27,28] and the amygdala and dopaminergic neurons play a major in responding to social rank (an individual’s social place as either subordinate or dominant in a group), and hierarchy signals [29]. Brain recordings indicate that loss of social status induces negative reward prediction error which via the lateral hypothalamus triggers the lateral habenula (anti-reward center), inhibiting the medial prefrontal cortex [30]. Using functional magnetic resonance imaging (fMRI), observing a powerful individual differentially engaged the dorsolateral prefrontal cortex, regions related to the amygdala (emotional processing), medial prefrontal cortex (social cognition) indicating a neural processing of social ranking and status in humans [31,32]. Furthermore, using fMRI, perceived social status was found to differentially modulate ventral striatal responses when processing social rank cues or status-related information [33]. Results from fMRI indicate that low social status is associated with diminished gray matter size in the perigenual area of the anterior cingulate cortex, which is associated with adaptive physiological, emotional, and behavioral reactions to psychosocial and environmental stressors [34]. Approach related motivation is linked to increased left-sided frontal activity in the brain, and the neural evidence of the relationship between approach related motivation and power was confirmed using EEG, which found that elevated power is connected with increased left-frontal activity in the brain compared to low power [35].

Also, power is linked with endocrinal and physiological changes. Testosterone increases dominance and other status-seeking behaviors [36,37] and this effect of testosterone on dominant behavior may be modulated by psychological stress and cortisol [38]. High testosterone has been identified as a factor that promotes the development of the socially destructive component of narcissism in powerholders [39], and power interacts with testosterone in predicting corruption [40]. Posing in high-power nonverbal displays causes physiological changes including increased feelings of power, a decrease in cortisol, increases in testosterone, and increased tolerance for risk compared to low-power posers [41]. Animal studies indicate that low social rank or subordination promotes stress activating the hypothalamic-pituitary-adrenal (HPA) and may modulate the brain’s dopaminergic function [42]. Multiple lines of evidence suggest that tryptophan enhances dominant behavior indicating that serotonin may promote dominance in humans [43,44]. Furthermore, results from experiments suggest that high social power elicits a benign cardiovascular response suggestive of a well-ordered cardiovascular pattern while low social power elicits a maladaptive cardiovascular response pattern which is suggestive of an inefficient cardiovascular pattern [45]. Power holders who may lose their privileged position displayed a maladaptive cardiovascular pattern, marked by low cardiac output (CO) and high total peripheral resistance which is suggestive of feeling threatened [46]. Evidence suggests that higher social status is associated with approach-type physiology compared to lower social status [47].

Power has a monumental effect on the behavior of the powerholder [2,48]. The corrupting effect of power is well known and has been a topic of interest for centuries to scholars. Plato advocated for the exclusion from office with consequential power, individuals who may misuse power for self-serving reasons, and only those with a well-developed sense of justice be allowed to wield power [49]. In recent decades, the corruption cases involving CEOs of large corporations, entrepreneurs, politicians, and autocrats/dictators have sparked both scholars’ and public interest in the corrupting effects of power [50–55] and this has triggered significant research into the effects of power on human behavior. Still, the full extent of power’s effect on behavior is not well understood. The monumental role that power plays in human interactions and life makes the need to better understand its effect on behavior both in powerholders and subordinates extremely important.

The objective of this paper is to elucidate the many corrupting effects of power or the need for power on human behavior as well as the structural limits of systems to hold powerholders accountable.

## 2. The corrupting effects of power or the need for power on human behavior

### 2.1. Power is addictive

There is evidence of addiction to the power derived from celebrity and fame [56]. The addictive effect on the powerholder promotes the need to engage in efforts to hold on to and accumulate power [57–59]. Aging, envy, and fear both conscious and unconscious of retaliation for previous acts may contribute to power’s addictiveness [58]. Efforts to hold on to power perpetually play a key in the practice of nepotism, factional struggle by powerful elites, cronyism, and dynastic succession [60–62].

Power abuse disorder has been coined as a neuropsychiatry condition connected to the addictive behavior of the power wielder [63]. Arguments have been made on the relationship between power addiction and dopaminergic alterations [63]. Indeed, changes in the dopaminergic system have been implicated in drug addiction [64] and research on animals suggests that dominance status modulates activity in dopaminergic neural pathways linked with motivation [27,28]. Evidence suggests that areas of the brain linked with addiction including the amygdala and dopaminergic neurons play a major in responding to social rank, and hierarchy signals [29]. Multiple lines of evidence from animal studies indicate that dopamine D2/D3 receptor density and availability is higher in the basal ganglia, including the nucleus accumbens, of animals with great social dominance compared to their subordinates [28,65,66]. Animal studies suggest that following forced loss of social rank, there is a craving for the privileges of status, leading to depressive-like symptoms which are reversed when social status is reinstated [30,67].

### 2.2. Power promotes self-righteousness, moral exceptionalism, and hypocrisy

Research indicates that powerful people are more likely to moralize, judge, and enforce strict moral standards on others while engaging in hypocritical or less strict moral behavior themselves [68]. In other words, powerful people often act and speak like they are sitting on the right hand of God to others especially subordinates while engaging in even worse unethical behavior. Being in a position of power with the discretion to apply punishment or reward to others allows the powerholder the freedom to do as they like or act inconsistently in so far as it serves their interests. This means powerholders are in a position to not necessarily practice what they preach with little or no consequences. Furthermore, being in a position to judge or take punitive action against others for their perceived moral failings may promote a false sense of moral superiority. This self-righteousness can create a misguided sense of probity and messianic zeal which can lead to poor decisions and outcomes. One takeaway from the relationship between power, self-righteousness, and hypocrisy is that power inhibits self-reflection or introspection.

This moral exceptionalism and hypocrisy also exist at the national and international levels. Powerful Western nations typically moralize and lecture about the rule of law, ethics, and democracy to other nations while hypocritically violating the same rules when it suits them or supporting allies that flagrantly violate the same rules [69–72].

Furthermore, mob action whether virtual or not is usually triggered by perceived injustice, a violation of societal norms, and unfair practices in the criminal justice system that undermine public institutional trust and confidence [73–76]. Placing wrongdoing on someone puts them (the wrongdoer) in a weaker power position socially which makes them vulnerable. With the power dynamics or balance tilted in the mob’s favor, the perceived injustice or wrongdoing envelopes the mob in an umbrella of sanctimony empowering them to act with impunity, and vigilantism by engaging in moral denunciations, bullying, destruction of property, and even lynching and other forms of violence toward the wrongdoer [77–79].

### 2.3. Power decreases empathy and compassion

Power decreases empathic concern [80] and is associated with reduced interpersonal sensitivity [81]. Research indicates that powerholders may experience less distress and less compassion as well as exhibit greater autonomic emotion regulation when faced with the pain of others [82]. Evidence indicates that elevated power impedes accurate understanding of other people’s emotional expressions [9,83] and is linked with poorer accuracy in emotional prosody identification than low power [84]. Elevated power is associated with heightened interest in rewards while low power is associated with increased attention to the interest of others [2,48,85].

Using transcranial magnetic stimulation, motor resonance which is the activation of similar brain pathways when acting and when observing someone act, implemented partly by the human mirror system was decreased in high-power holders relative to low-power holders [81]. Evidence suggests a linear relationship between the motor resonance system and power in which increasing accumulation of power is connected to decreasing levels of resonance [81]. This change might be one of the neural mechanisms that underlie power-induced asymmetries in social interactions [81].

Also, higher socioeconomic status is associated with reduced neural responses to the pain of others [86,87]. In contrast, a lower socioeconomic level is associated with higher compassion, being more attuned to the distress of others [88,89] and more empathically correct in evaluating the emotions of other people [90] compared to upper-socioeconomic class. High status is associated with exhibiting less communal and prosocial behavior and decreased likelihood of endorsing more egalitarian life goals and values compared with those with low status [91]. In addition, higher-class people are more likely to endorse the theory that social class is steeped in genetically based (heritable) innate differences than lower-class people and display reduced support for restorative justice [92].

### 2.4. Power promotes disinhibited behavior and overconfidence

Elevated power is associated with disinhibited behavior, increased freedom, and heightened interest in rewards while low power is associated with inhibited social behavior [2,48,85]. Power is associated with optimism and riskier behavior [93] and it enhances self-regulation and performance [94]. It energizes, speech, thought, and action and magnifies confidence, and enhances self-expression [14,25]. Power elevates self-esteem and impacts how people evaluate and view themselves in comparison to others [25,95]. Elevated power particularly in narcissistic individuals results in significant overconfidence compared to individuals in a low state of power [96].

Power increases the illusion of control over outcomes that are outside the reach of the powerholder [97]. It distorts impressions of physical size with the powerful exaggerating their height and feeling taller than they actually are [98], underestimating the size of others, and the powerless overestimating the size of others [99].

### 2.5. Power promotes unethical behavior and entitlement

Power promotes feelings of entitlement [100] and powerholders are not often cognizant of their violation of basic fairness principles [25]. Evidence from experiments using fMRI indicates that power promotes greed by increasing aversion to receiving less than others and reducing aversion to receiving more than others [101]. Powerholders, particularly pro-self-individuals, displayed decreased response in the right and left dorsolateral prefrontal cortex, indicating a weaker restrain of self-interest when processing receiving more than others [101]. The need for power is significantly and positively correlated with narcissism [102,103]. Power amplifies the tendency of self-focused goals to result in self-interested behavior [104] and may cause people to act unethically in their self-interest [50–52,105]. Powerful people tend to move in the same circles, giving them access, and increased likelihood of having relationships with other powerful people and these relationships may foster unethical behaviors including quid pro quo, nepotism/favoritism, cronyism, mutual protection against threats, ignoring or bypassing of due process, conflict of interests and corruption.

Physical attractiveness influences people’s social evaluations of others and attractive people enjoy benefits in terms of perceived good health, power, economic advantage, confidence, trust, perceived intelligence, and popularity [106–112]. Research suggests that the power of perceived attractiveness is associated with increased self-interested behavior and psychological entitlement [113]. Furthermore, power gained from improved physical appearance/attractiveness, increased attention, improved self-image, and self-confidence following bariatric surgery weight loss is linked to increased separation/divorce [114–116]. This suggests that power from improved physical appearance and attention following bariatric surgery may promote entitlement, narcissism, and self-interested behavior.

Power makes powerholders feel special, invincible, and above the rules. Indeed, car cost predicts driver yielding to pedestrians with more expensive car drivers less likely to yield to pedestrians at a crosswalk [117]. While driving, individuals of higher-class are more likely to break the law compared to lower-class individuals and are more likely to cheat and lie and display unethical decision-making tendencies than lower-class individuals [118].

### 2.6. Power promotes aggressive and dehumanizing behavior

Power promotes dehumanization, which is the process of rejecting essential components of “humanness” in others and seeing them as animals or objects [119,120] while powerlessness leads to self-dehumanization [121]. Power promotes the objectification of others [122] and increases the tendency to disparage and engage in harmful behavior toward others including bullying, autocracy, and manipulation [123–125].

Also, elevated power is associated with manipulative and contemptuous behavior toward people with low power by devaluing their worth [126]. It is associated with demeaning, and dehumanizing behavior toward others with low power, with more power resulting in more demeaning behavior [127,128]. Notably, individuals in high power but lacking in status (e.g., prison guards, soldiers) display increased interpersonal conflict and demeaning behaviors [127,129]. Furthermore, research indicates that a powerholder’s threat assessment elicits escalation or confrontational behaviors toward subordinates and de-escalation or submissive behaviors toward higher-status or dominant superiors [130]. In defense of their ego, power coupled with feelings of incompetence can promote aggressive behavior [131].

One key reason for the emergence of this demeaning and dehumanizing behavior of powerful people is their false sense of superiority over individuals with low power. This is reinforced by the excessive praise and groveling of subordinates and the fact they are they have the authority to impose negative consequences on others, and few are bold enough to challenge them out of fear of retaliation. This feeling or sense of superiority is particularly more pronounced in an environment where there is little to no oversight over their behavior, and it can gradually divorce them from reality. Jokes that were once considered mundane or innocuous before they acquired power or accumulated more power are suddenly perceived as insults. Anyone who dares to argue for a different position, especially one that suggests incompetence, is perceived as a threat that needs to be eliminated.

Moreover, experimental evidence indicates that asymmetric power differences can promote extortionary [132] and exploitative behaviors [133]. The power asymmetry between human traffickers and the young, vulnerable people they exploit explains the sense of entrapment of survivors, why the traffickers can engage in dehumanizing and demeaning behavior, violence, and forced labor with impunity, without any sense of guilt, remorse, or regard for the welfare of the trafficked individuals [134–136]. The power asymmetry between police officers and vulnerable people in their community (e.g., sex workers, the homeless, marginalized people, and minorities) explains to some extent the increased likelihood of police abuse toward members of those communities [137–139]. There are many stories of seemingly normal people enslaving and using violence against their maids [140,141]. Usually, people who become trapped in these situations are foreigners with no legal documentation or with legal papers connected to their work for that employer. The significant asymmetric power difference between the employer and the maid makes the maid vulnerable to abuse. Anyone in the position of employer can easily become abusive toward the vulnerable maid in an environment where negative consequences for their actions are nonexistent.

This same power asymmetry which may lead to bullying, intimidation, and exploitation can be observed between nation-states. Just like individuals, as disparities in economic and military power widen between countries, the larger and more powerful states may engage in bullying neighboring states through trade and other means including threats of war if they act outside of ways the more powerful nations prefer.

### 2.7. Power sexualizes social interactions

Power is linked with sex [142]. It elicits romantic desire from individuals of the opposite sex [143] and may play an important role in sexual objectification [144,145]. Evidence suggests that subordinates view their leaders as significantly more physically attractive [146] and power increases expectations of sexual interest from subordinates biasing social judgment and sexualizing social interactions which might lead to sexual harassment [147].

Power is positively associated with sexual infidelity because of its disinhibiting effects on behavior and increased self-confidence to attract partners [148,149]. Its disinhibiting effect also amplifies the appetite for both normative or counter-normative forms of sexuality and makes powerful men seem more desirable and attractive which may increase their access to potential sexual opportunities [148]. Power asymmetry between educators and students increases the potential for sexual misconduct and abuse [150–153].

Boundary setting, vigilance, and regular training for teachers and organizational supervisors on the sexualizing effect of power on social interactions should be put in place to reduce the incidence of sexual harassment and inappropriate relationships.

### 2.8. Power hinders perspective taking and cooperation

Low power is associated with increased cooperation [154] while elevated power may hinder perspective-taking [83] and increase the preference for the preservation of psychological distance from people with low power [126,155]. An fMRI study showed that powerholders display reduced neural activation in regions associated with cognitive control and perspective-taking (frontal eye field and precuneus) [101]. Results from electroencephalogram (EEG) suggest that power taints balanced cooperation by reducing the power holder’s motivation to cooperate with subordinates [156]. Also, power reduces conformity to the opinion of others [9,157] and is associated with discounting advice, due to overconfidence [158–160] as well as being less trusting [161] and this can hamper cooperation.

### 2.9. Power, judgment bias, and selective information processing

Power promotes the need for less diagnostic information about others and increases vulnerability to using preconscious processing and stereotypical information about others [162–165]. It increases implicit prejudice (racial bias) and implicit stereotyping [166,167]. Evidence suggests that elevated power is associated with automatic information processing, while low power is associated with restrictive information processing [2,48,85]. Power modulates basic cognition by promoting selective attention to information and suppressing peripheral information [168]. Results from an experiment found that neural activity in the left inferior frontal gyrus, an area linked with cognitive interference, was diminished for individuals with elevated power relative to those with low power suggesting that elevated power may reduce cognitive interference [169].

Elevated power promotes social attentional bias toward low-power holders [170]. It also promotes self-anchoring attitudes, traits, and emotions which is the use of the self as the gold standard or reference point for evaluating or judging others [171]. In other words, for powerful people good or bad traits and attitudes are viewed using themselves as a reference without regard for the individuality of others. Power modulates the process of making tough decisions [172] and it is associated with excessive confidence in judgment which may turn out to be less accurate [158–160].

### 2.10. Power confers credibility

Credibility carries power and power confers credibility relative to those with less power [173,174]. The claims or assertions of a person with power or high status are typically treated with respect. In contrast, the claims of individuals at the lower end of the power structure are often doubted until investigated, and that is if anyone even bothers to investigate thoroughly and fairly. Consider the Filipino maid working in Kuala Lumpur, the Ethiopian or Indian lady working as a maid somewhere in the Middle East, or the young girl from Calabar working as a maid for a rich family in Lagos. Typically, maids depend on their employers not just for housing and food, but for their immigration status as well. Who will believe her if she accuses her boss of sexual assault or if her boss falsely accuses her of stealing? Similarly, if a police officer, particularly one with an unblemished record, plants drugs on an ex-convict, who is going to believe the ex-convict? The more he protests, the guiltier he appears.

In the workplace, the significant power asymmetry between an employee and their supervisor gives their supervisor significant credibility. A report from a supervisor, whether true or false, carries considerable weight because of the credibility they automatically have relative to their employee.Disturbingly, the supervisor’s powers do not end within the four walls of the organization; employers at other organizations may depend on the assessment and opinion of the supervisor to pass judgment on a person without any regard for the possibility of their prejudice.

### 2.11. Power and victimhood

Not all victims are after power but being a victim can come with significant power [175–179]. Victims are seen as socially and morally superior and deserving of social deference [180,181]. Victimhood proffers psychological and social benefits and allows one to achieve greater social or political status [181,182]. This makes victimhood attractive.

The need for power significantly predicts competitive victimhood, which is a tendency to see one’s group as having dealt with more adversity relative to an outgroup [177–179]. Victims, especially those who appear weak or who are lower in the power structure, are seen as needing protection. In contrast, the accused are seen as aggressive and dangerous. The power derived from victimhood can be misused, and many people employ it for retribution. Being a victim or feeling wronged may result in a sense of entitlement and selfish behavior [182].

While it is important to protect victims in all cases, care must be taken to ensure that negative consequences are not applied reactionarily to the accused. Negative actions taken against the accused before a fair and thorough investigation is conducted make the exploitation of victimhood attractive. Even if the allegations are proven to be false, public outrage and adverse opinion can lead to irreparable reputational damage and financial loss. The noble pursuit of an equal and fair society must never blind us to the dangers posed by the exploitation of the power of victimhood to elicit outrage and pursue retribution.

### 2.12. Power and gossip

Gossip tends to be negative, and people engage in it for many reasons including for socializing, to gain influence and power, due to perceptions of unfairness, feelings of envy, jealousy, and resentment, to get moral information, creation and maintenance of in-groups and out-groups, indirect aggression, and social control [183–186]. Gossip has self-evaluative and emotional consequences [187].

Spreading gossip can be an effort to exercise power [188]. Lateral gossip or gossip between peers of similar power can help people get information and support from others. However, upward gossip which is gossip with people in higher power who have formal control over resources and the means to take action may be used by those in lower power to inform and thereby gain or exert influence [189]. Reputation and gossip are intertwined, and gossip can be used for status enhancement and wielded as a weapon against others [190].

The need for power may cause people to engage in gossiping and a person with a listening and believing audience of one has the power to destroy another person’s reputation and adversely affect their life.

### 2.13. Power and ambition

Ambition, defined as the persistent or relentless striving for success, attainment, and accomplishment or a yearning desire for success that is committedly pursued [191], is crucial to success in diverse social contexts. Ambition is positively associated with educational attainment, high income, occupation prestige, and greater satisfaction with life [192,193]. Power and ambition are inextricably linked because people with power and those who aspire for power are typically very ambitious. Ambition is critical in acquiring, accumulating, and retaining power.

Ambition, while critical to being successful [193,194] and an immensely powerful motivator, can also be a potent self-destructive tool and a vice that may cause people to inflict suffering on others in the pursuit of personal glory and gains [191]. Overreaching ambition breeds greed and can quickly slip into dishonesty [195,196]. Ambition and greed encourage both destructive competition and acquisitiveness as a way to affirm superiority over others [197]. Excessive ambition can be a curse as it can lead to extremism due to obsessive passion [198] and make people feel dissatisfied even with their accomplishments because their desires are insatiable or can never be fully achieved [191,199]. Ambition can make a person falsely believe that they are special, destined for greatness, or cut from a different cloth. While this feeling can be helpful in the pursuit of seemingly challenging goals, it can lead to unethical behavior [195,200].

In efforts to retain power and status, ambition can make people abuse power and for those trying to acquire power, it can make them go to extra lengths without regard for the negative consequences. Indeed, excessive ambition in powerful people or excessive ambition for power, fame, and prestige can blur the lines of acceptable behavior, and when those lines are crossed, it can result in actions that are fraudulent, illegal, and catastrophic [53,201–204]. Ambition can cause a person to act recklessly by exaggerating both reality and possibilities, as well as by downplaying important risks that may prove fatal. When people begin to see the end goal as the only thing that matters, they cut corners, and lose sight of ethics and the monumental danger their actions pose to others. In line with the dangers of ambition, Machiavelli argued that ambition and greed are the causes of chaos and war [197].

## 3. Power, and the structural limits of accountability systems

In most social systems, people who are lower in the power structure can only get misconduct addressed by a third party that has some power to punish, hold accountable, or overturn the judgment imposed by the powerholder. For example, an employee with allegations of wrongdoing by their manager, who is the CEO or President of the organization may not be able to hold them accountable within the organization. Their case may be best addressed by the court system, a third party with the authority to hold the organization accountable. Seeking fair redress or accountability within the organization can be difficult or even impossible because those in power are not motivated to change their behavior. So, unless the employee is willing to take their case to court (or another authority with a similar power to hold the employer accountable, like the press), there may not be a way for them to seek redress. Unfortunately, a third party is often not present, and even if one exists, it may not be impartial or easily accessed by people lower in the power structure.

Furthermore, there is a limit to the number of third parties or higher authorities in any social system for seeking redress. At some point, there must be a supreme authority whose ruling is final and irreversible. In a nation-state, the final authority may be the apex or Supreme Court. In sports, a ruling body makes final decisions. In the global arena, international courts have the final say against individuals or nations that violate relevant laws. Importantly, if the judgment of the top authority is incorrect or unjust, the only option is to accept the ruling until the issue is revisited. Also, the higher you must go in efforts to seek redress for wrongdoing, the less accessible it is for people who are lower in the power structure, and the fewer cases that are worthy of being taken on. These obstacles mean that many cases of power abuse go unchecked, unfair judgments are often passed, and miscarriages of justice occur at all levels. In addition, falsehoods about people and events sanctioned or protected by the powerful are carried as truth into posterity.

So, the means for holding accountable or checking the actions of the powerful by those with low power are limited not just by corruption and problems of access but by the structural limits of accountability/justice systems.

## 4. Discussion

The role of power in our lives is all-pervasive, and complex, and its effects extend to both intentional and unintentional acts of the powerholder [4]. The current review is different from previous works and contributes significantly to our understanding of power because of its extensiveness and broad synthesis of the literature on power from a wide range of disciplines including biology, neuroscience, psychology, behavioral sciences, sociology, and anthropology. One key lesson from this work is that the effects of power extend beyond the behavioral changes that are visible as power interacts with the neurological, neuroendocrine, psychological, and physiological processes of the power holder.

**Figure 1.**
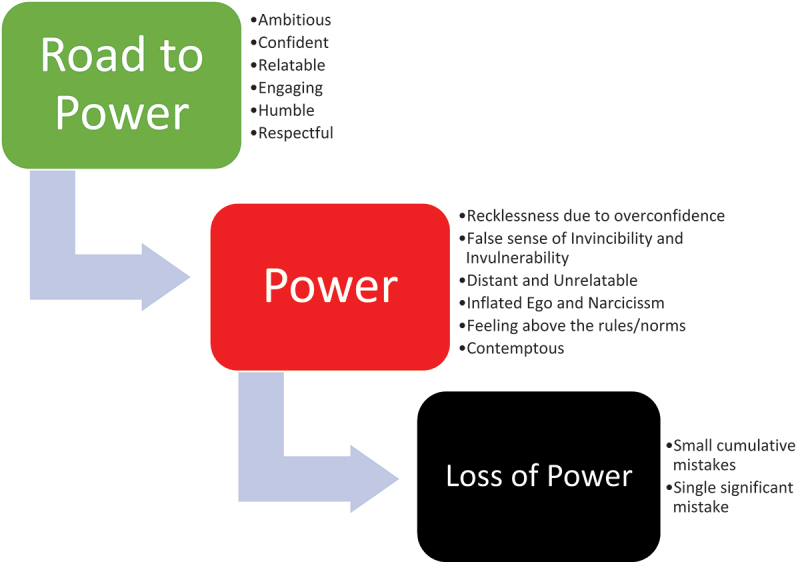
Classical process of power corrupting behavior leading to power loss.

**Figure 2.**
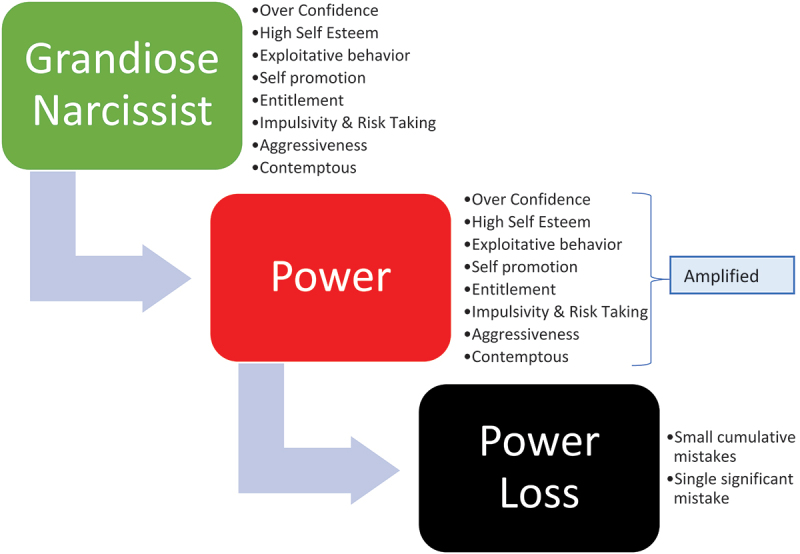
Narcissist model of power corrupting behavior leading to power loss.

As noted in Figure 1, power can dramatically change ordinary people’s behavior causing them to abuse it thereby making cumulative small mistakes that reach a dangerous threshold or a single significant mistake that ultimately leads to their loss of power. The narcissist personality model described in Figure 2 is different from the classical Model (Non-narcissist). The grandiose narcissist is assertive and extraverted and distinguished by their sense of entitlement, overconfidence, high self-esteem, feelings of personal superiority, self-serving exploitative behavior, impulsivity, a need for admiration and dominance, and aggressive and hostile behavior when threatened or challenged [205–208]. Grandiose narcissists are more likely to seek and achieve positions of power in organizations [209–213], but they are more likely to abuse their power, pursue their interests at the expense of the organization [207,214–217], disregard expert advice causing them to make poor decisions [205].

Another key takeaway from this paper is that no human being is completely immune to the corrupting effects of power. Results from a lab experiment suggest that power amplifies people’s dispositions in which powerful people with a firm moral identity are less likely to act in self-interest relative to those with a shaky moral identity [105]. One argument against the conclusions of this experiment is that power roles in lab experiments typically do not involve consequential outcomes or real decisions [4] and may not translate to power experiences in the real world [5]. Furthermore, the effects of power may change when it involves genuine interpersonal interactions compared to the arbitrary assignment into power groups, hypothetical scenarios, or anticipated interactions, as in a lab [5]. Another argument against this conclusion is the evidence that the virtue of honesty may not protect powerful people from the corruptive effect of power (Bendahan et al., 2015), Even with a strong moral identity, exposure to cash can provoke unethical intentions and behavior [218]. Even with a strong moral identity, it is still possible that in the presence of a threat to ego or power, seemingly good people with power can abuse power by acting aggressively [104,131,219]. Evidence suggests that in efforts to avoid a status or power loss powerful people may be willing to use coercion and go extra lengths even at others ‘expense [104,219,220]. Also, appetitive aggression, the nature of lust for violence, is an innate part of human behavior [221] and humans by nature have a high propensity for proactive aggression, a trait possessed in common with chimpanzees [222]. Indeed, human hands are evolved for improved manual dexterity and to be used as a club during fighting [223]. The neurobiology of human aggressive behavior has been extensively studied and includes alterations in brain regional volumes, metabolism, and connectivity in certain neural networks. Subregions of the prefrontal cortex, amygdala, insula, hippocampus, and basal ganglia play a critical role within these circuits and are linked to the biology of aggression [224]. So, while there are individual differences in propensity to abuse power including the use of violence and aggression [225], the monumentally corrupting effects of power can ensnare anyone. Taken together, when it comes to power, there are no good or bad people, there are only people.

Organizational social hierarchies play an important role in power abuse. Power hierarchies and pyramidal forms of leadership are integral aspects of social organizations to help create stability and order, but they attract narcissistic individuals [226] and can be harmful [227]. In many cases, these hierarchical structures can perpetuate power differences, creating bureaucratic conditions where there are strictly defined roles, with their distinction and importance overstressed. Being an individual with low power in such an environment can be challenging because of powerlessness and powerlessness can lead to self-dehumanization and feelings of worthlessness [121]. Such an environment can also stymie creativity, particularly for people with low power. Indeed, several lines of evidence indicate that power increases creativity [155,157,228,229]. However, when the power hierarchy is not fixed, people with low power display a flexible processing style and greater creativity [230]. So, organizations need to use a mixed model of classical hierarchy that incorporates flat hierarchy as much as possible to ensure that all members feel empowered and have a strong sense of belonging. Notably, an environment where people with low power feel empowered may result in decreased temporal discounting and increased lifetime savings [231]

It is important to note that there are some valid explanations for some of the behavior that powerholders display. Indeed, powerful people may pay less attention and be more vulnerable to stereotyping because they are attentionally overloaded leading to scarce cognitive resources [4,163]. Power is associated with a greater feeling of responsibility, and this may explain to some extent why it is associated with reduced social distance [5] Also, there are conflicting reports in the literature regarding the corrupting effect of power on behavior. Power used corruptly may play a vital role in maintaining cooperation in human society [8,232]. Power may not promote intransigence instead it can create internal conflict and dissonance leading to a change in attitude [157]. Instead of creating social distance, elevated power has been found to be associated with attentiveness in interacting with other people and greater feelings of being close to them relative to low power [5]. Experimental evidence suggests that high power is associated with more interpersonal sensitivity than low power [233]. Furthermore, high-status individuals have been found to display more prosocial behavior and to be more generous, trusting, and trustworthy compared to low-social-status individuals [234]. Power has been found to have no effects on attraction to rewards, which runs counter to the approach/inhibition theory that suggests that power enhances individuals’ interest in rewards [235]. Also, experimental evidence indicates that power under certain circumstances can result in less risky or more conservative behavior [236]. These findings indicate that more studies are needed to better understand the effects of power using better experiment designs with larger samples and more real-world studies. It also indicates that power abuse mitigating factors can play a critical role in curbing the corrupting effects of power.

The keys to maintaining and being effective with legitimate power are understanding its corrupting effects, continued relatability, collaboration, respect for peers and subordinates, and humility, which is predictive of positive outcomes [237]. The corrupting effect of power makes the need for checks and balances important to ensure the proper functioning and success of all individuals of a social group. One of the ways of mitigating power abuse is the consideration of predispositions, proper vetting to select ethical candidates, and training to increase social responsibility in people appointed to positions of power [25]. Organizational culture can play an important role in mitigating power abuse as it can shape and nurture power holders through values and culture that link power with being responsible [238]. Appropriate negative consequences must be put in place to deter the abuse of power. More must be done in the selection and training of individuals with power over highly vulnerable people with low power from abuse e.g., children, the institutionalized, etc. Physicians have power over patients in many respects [239,240] and the trend toward shared decision-making [241] must be strengthened using medical education training of physicians in the appropriate use of power and enactment of patient-centered therapeutic communications [242]. Boundary setting, vigilance, and regular training for teachers and organizational supervisors on the sexualizing effect of power on social interactions should be put in place to reduce the incidence of sexual harassment and inappropriate relationships. To mitigate the negative effects of the structural limits of accountability systems, allegations of wrongdoing by the powerful should be treated seriously and everyone particularly those in the lower power structure should be guaranteed access and resources to a fair and impartial higher authority for addressing wrongdoing without fear of retaliation. The allowance and development of a robust civil society that can leverage the power of peaceful protests to bring about change are crucial to pushing back on the excesses of power. The continued promotion of universal human rights and the creation of international institutions that hold powerful people accountable for blatant abuse of power is another important tool to deter and reduce the incidence of blatant abuses of power. In the international arena, laws and governing bodies must protect smaller nations from bullying, intimidation, and threats from larger and more powerful nations.

Finally, while intoxicating, power is fleeting, and it goes around. A person with immense power today may be lacking in power tomorrow. In the same vein, a person with little relevance today could ascend to a position of great power tomorrow. This should serve as a warning to everyone with power: always treat others with dignity, respect, and compassion, regardless of their current place in the power structure. As they say, the future is pregnant, and no one knows exactly what it will deliver.
